# Phase transformation and mechanical properties of heat-treated nickel-titanium rotary endodontic instruments at room and body temperatures

**DOI:** 10.1186/s12903-023-03550-6

**Published:** 2023-10-30

**Authors:** Yuka Kasuga, Shunsuke Kimura, Keiichiro Maki, Hayate Unno, Satoshi Omori, Keiko Hirano, Arata Ebihara, Takashi Okiji

**Affiliations:** Department of Pulp Biology and Endodontics, Division of Oral Health Sciences, Graduate School of Medical and Dental Sciences, Tokyo Medical and Dental University (TMDU), 1-5-45 Yushima, Bunkyo-ku, Tokyo, 113-8549 Japan

**Keywords:** Bending property, Cyclic fatigue resistance, Differential scanning calorimetry, Nickel-titanium rotary instrument, Phase transformation, Root canal instrumentation

## Abstract

**Background:**

The aim of this study was to evaluate the phase composition, phase transformation temperatures, bending property, and cyclic fatigue resistance of different heat-treated nickel-titanium (NiTi) rotary instruments with the same tip diameter and taper at room (RT; 25 ± 1 °C) and body (BT; 37 ± 1 °C) temperatures.

**Methods:**

Five heat-treated NiTi rotary instruments, HyFlex EDM (EDM), HyFlex CM (CM), Vortex Blue (VB), RE file CT (RE) and JIZAI, and a non-heat-treated NiTi rotary instrument (Mtwo) with a size 40, 0.04 taper were investigated. Temperature-dependent phase transformation was examined with differential scanning calorimetry (DSC). The bending loads of the instruments at RT and BT were evaluated using a cantilever-bending test. Cyclic fatigue resistance at RT and BT was measured using a dynamic test, during which the instruments were rotated in combination with a 2-mm back-and-forth motion in an artificial curved canal, and the number of cycles to failure (NCF) was determined. The results were analyzed using two-way repeated measures analysis of variance, a simple main effect test, and the Bonferroni test (α = 0.05).

**Results:**

DSC results indicated that EDM and Mtwo were primarily composed of martensite/R-phase and austenite, respectively, while the other heat-treated instruments were composed of a mix of martensite/R-phase and austenite at the tested temperatures. Regardless of the temperature setting, the bending loads of heat-treated instruments were significantly lower than those of Mtwo (*p* < 0.05). EDM showed the lowest bending loads and highest NCF at both temperatures (*p* < 0.05). CM, VB, and JIZAI showed significantly higher bending loads at BT than at RT (*p* < 0.05). The NCF of all the heat-treated instruments, except VB, was lower at BT than at RT (*p* < 0.05). At BT, the NCF of CM, VB, RE, and JIZAI were not significantly higher than that of Mtwo (*p* > 0.05).

**Conclusions:**

Heat-treated NiTi instruments exhibited lower bending loads and higher NCF values than Mtwo. However, this tendency was less pronounced at BT than at RT, especially in the NCF values of instruments with a mixture of martensite/R-phase and austenite phases at the tested temperatures.

## Background

Nickel-titanium (NiTi) rotary endodontic instruments have gained considerable popularity because their flexibility, canal-centering ability, and cutting efficiency are superior to those of stainless steel hand instruments [1, 2]. However, intracanal fracture of these instruments is a concern that remains unsolved [3–5]. Thus, manufacturers have developed various new instruments to exhibit improved fracture resistance by modifying the geometry, metallurgy, and manufacturing processes [6, 7]. However, the mechanical properties of these new instruments need to be gathered to enable informed selection of instrument(s) for optimal safety and predictable treatment results.

NiTi alloys exhibit superelasticity and shape memory owing to the reversible transformation of their phases: martensite, R-phase, and austenite. For example, NiTi alloys become superelastic at temperatures above the austenite (reverse transformation) finishing temperature (A*f*), which is the temperature at which transformation to austenite is complete [8]. At temperatures below A*f*, NiTi alloys are rich in martensite and R-phase, which are less rigid and more ductile than austenite [9, 10]. Thus, temperature-dependent phase transformation determines the phase composition and thus the physical property of NiTi alloys. The temperature-dependent crystallographic state of NiTi alloys is influenced by the atomic composition, manufacturing processes, and treatment conditions of NiTi alloys [11]. Among these, heat treatment is widely applied to raise A*f* and increase the proportions of martensite and R-phase, which enhances the flexibility of NiTi rotary instruments at clinical temperatures [12–14]. This process varies among available NiTi rotary instruments, and plays an important role in determining their mechanical properties at different environmental temperature [14, 15]. Therefore, characterization of the temperature-dependent phase composition of these instruments is crucial in understanding their physical performance.

The mechanical properties of heat-treated NiTi rotary instruments are usually investigated at room temperature (RT) [15–18], which may limit the clinical relevance of the results owing to the altered phase composition. In particular, the cyclic fatigue resistance of some heat-treated NiTi rotary instruments is higher at RT than at body temperature (BT) because the austenite proportion is greater at BT than at RT [19, 20]. The temperature-dependent phase composition also influences flexibility [21], which is among the critical factors determining cyclic fatigue resistance [20, 22]. However, few studies have investigated whether and how different temperature conditions affect the cyclic fatigue life and bending properties of heat-treated NiTi rotary instruments, which are related to phase transformation.

The geometry of NiTi rotary instruments is an essential factor determining their mechanical properties [23]. In particular, instruments with a wider cross-sectional area exhibit lower cyclic fatigue resistance than those with a narrower cross-sectional area [23, 24]. However, most studies comparing commercially available instruments suffer from a major drawback, which is the inability to standardize geometrical factors among the instruments employed. For example, several studies have shown that HyFlex EDM (EDM; Coltene-Whaledent, Allstätten, Switzerland) has high flexibility and cyclic fatigue resistance [13, 16, 23, 25–27], although the unique variable taper of this instrument may be regarded as a confounding parameter.

The purpose of this study was to evaluate the design, phase transformation temperatures, bending property, and cyclic fatigue resistance of six brands of differently heat-treated NiTi rotary instruments at RT and BT. To reduce geometrical confounding parameters, size- and taper-matched instruments were investigated. The following null hypotheses were tested: There are no differences in (i) bending load values and (ii) cyclic fatigue life among the six brands of instruments and for each brand of instrument at RT and BT.

## Methods

### Instruments

Five brands of heat-treated NiTi rotary instruments, Hyflex EDM (EDM; Coltene-Whaledent, Altstätten, Switzerland), HyFlex CM (CM; Coltene-Whaledent), Vortex Blue (VB; Dentsply Tulsa Dental Specialties, Tulsa, OK, USA), RE file CT (RE: Yoshida Dentcraft, Tokyo, Japan), and JIZAI (MANI, Tochigi, Japan), and a non-heat-treated NiTi rotary instrument (Mtwo; VDW, Munich, Germany) were selected for this investigation. All instruments were size 40 with a taper of 0.04.

### Instrument geometry

One instrument in each brand was randomly selected, embedded in an epoxy resin (Epofix; Struers Aps, Ballerup, Denmark), and cut at 7 mm from the tip. Each cross-section was examined with a confocal laser scanning microscope (OLS4000, Olympus, Tokyo, Japan; × 216 magnification). The cross-sectional area and the core diameter was calculated using image analyzing software (Photoshop Elements 2021, Adobe Systems, San Jose, CA, USA). Longitudinal images of the instruments were taken using a digital microscope (VH-800, Keyence, Osaka, Japan; × 20 magnification), and the number of blades from the base to the tip of the cutting portion were counted.

### Sample size estimation

The required sample was determined using the G*power software (version 3.1.9.7, Heinrich Heine Universität, Düsseldorf, Germany). A priori analysis of variance (ANOVA; fixed effects, special, main effects and interactions) was selected from the F-test family. Based on an effect size of 0.4, alpha-type error of 0.05, and power beta of 0.85, a previous study with similar methodology [16] required a minimum sample size of 8 per group to reveal statistical significance in terms of bending load and dynamic cyclic fatigue values. Therefore, a sample size of 10 per group was used for bending test and dynamic cyclic fatigue test.

### Differential scanning calorimetry

Differential scanning calorimetry (DSC) was performed to investigate the phase transformation of the NiTi alloys. Specimens (*n* = 5 in each instrument group), taken from the blade portion of an instrument, were cut into 2- to 3-mm-long segments weighing 20 mg and enclosed in the aluminum cells of a differential scanning calorimeter (DSC-60, Shimadzu, Kyoto, Japan). Measurements were made in the temperature range of + 100 to − 100 °C under an argon gas atmosphere. Liquid nitrogen was used as the coolant, and the heating and cooling rates were set to 10 °C/min. Alpha-alumina powder (20 mg) was also enclosed in an aluminum cell and used as the reference material. The following transformation temperatures were determined from the DSC curves obtained for each specimen: martensitic transformation starting temperature (M*s*), martensitic transformation finishing temperature (M*f*), reverse transformation starting temperature (A*s*), A*f*, R-phase transformation starting temperature (R*s*), and R-phase transformation finishing temperature (R*f*). Interpretation of the DSC curves was based on previous studies [14, 15, 28].

### Bending test

The cantilever-bending test [14–16] was conducted at RT (25 ± 1 °C) or BT (37 ± 1 °C) in an incubator (RKC Instrument Inc, Tokyo, Japan). Briefly, the instruments (*n* = 10 each) were fixed 7.0 mm from the tip, loaded at 1.0 mm/min to a position 2 mm from the tip until the displacement reached 3.0 mm, and then unloaded. Bending load values were evaluated at a deflection of 0.5 mm and 2.0 mm, corresponding to the elastic and superelastic ranges, respectively [14].

### Dynamic cyclic fatigue test

The dynamic cyclic fatigue test [16] was conducted at RT (25 ± 1 °C) or BT (37 ± 1 °C) in an incubator made of a plastic box and a temperature controller (E5C4, OMRON Corporation, Kyoto, Japan). The testing device consisted of a test stand with a movable stage (MH2-500N, IMADA, Aichi, Japan), to which the handpiece of an endodontic motor (Tri Auto ZX2, J. Morita, Kyoto, Japan) was attached. A 17-mm-long stainless steel artificial canal with a 1.5-mm diameter, 60° angle of curvature, and 3.0-mm radius of curvature was used, and the environmental temperature of the canal was checked with a thermocouple (HOBO UX120-014 M, Onset Computer Corporation, Bourne, MA, USA). The center of the canal curvature was located 5 mm from the tip of the instrument. The instruments (*n* = 10, each) were fixed in the canal at 13.6 mm and rotated in combination with a 2-mm back-and-forth motion at 5 mm/s. Silicone oil (KF-96-100CS, Shin-Etsu Chemical, Tokyo, Japan) was used as lubricant. The rotational speed was set to 300 rpm for Mtwo and 500 rpm for EDM, CM, VB, RE, and JIZAI, according to the manufactures’ recommendation. The time to fracture was recorded using a stopwatch, and the number of cycles to failure (NCF) was calculated as rpm × time to failure (min).

### Statistical analysis

Normality and variance homogeneity of the values obtained in the bending test and dynamic cyclic fatigue test were verified using the Shapiro–Wilk test and Levene's test, respectively. A two-factor factorial analysis of variance (ANOVA) was performed with temperatures and instruments as independent variables and NCF or bending loads as dependent variables, and the main effect and interaction were analyzed. If the interaction was significant, a simple main effect test was conducted, and the Bonferroni test was used for multiple comparisons. A *p*-value of < 0.05 was considered statistically significant.

## Results

### Instrument design

**Fig. 1:**
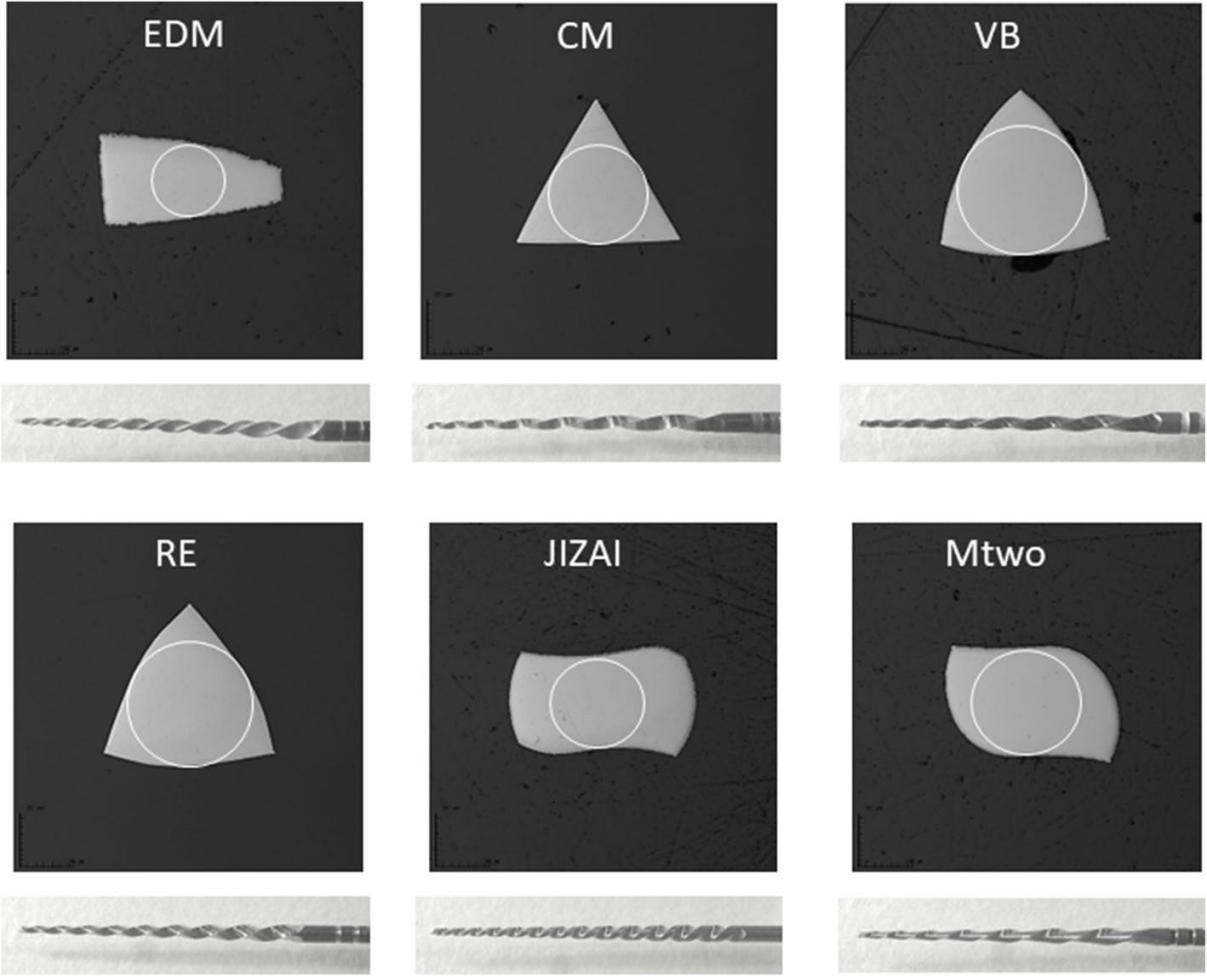
Cross-sectional and lateral views of the cut portion of tested instruments. Cross-sectional images are taken at 7 mm from the tip. Circles show the inner core of each instrument

Figure 1 presents the cross-sectional and lateral images of each tested instrument. The instruments differed in cross-sectional shape, except VB and RE, which shared a convex triangular cross section. As shown in Table 1, EDM and CM had smaller cross-sectional areas than the other instruments.

**Table 1:** Geometric parameters of NiTi instruments tested in this study

Instrument	Core diameter (µm)	Cross-sectional area (mm^2^)	Cross-sectional shape	Number of blades
EDM	279	0.174678	Trapezoidal	16
CM	375	0.170429	Triangular	7
VB	481	0.249002	Convex triangular	9
RE	481	0.244369	Convex triangular	10
JIZAI	362	0.250714	Rectangular, radial-landed	13^a^
Mtwo	423	0.240731	S-shape	8

^a^Number of radial lands

### Phase transformation temperatures

**Fig. 2:**
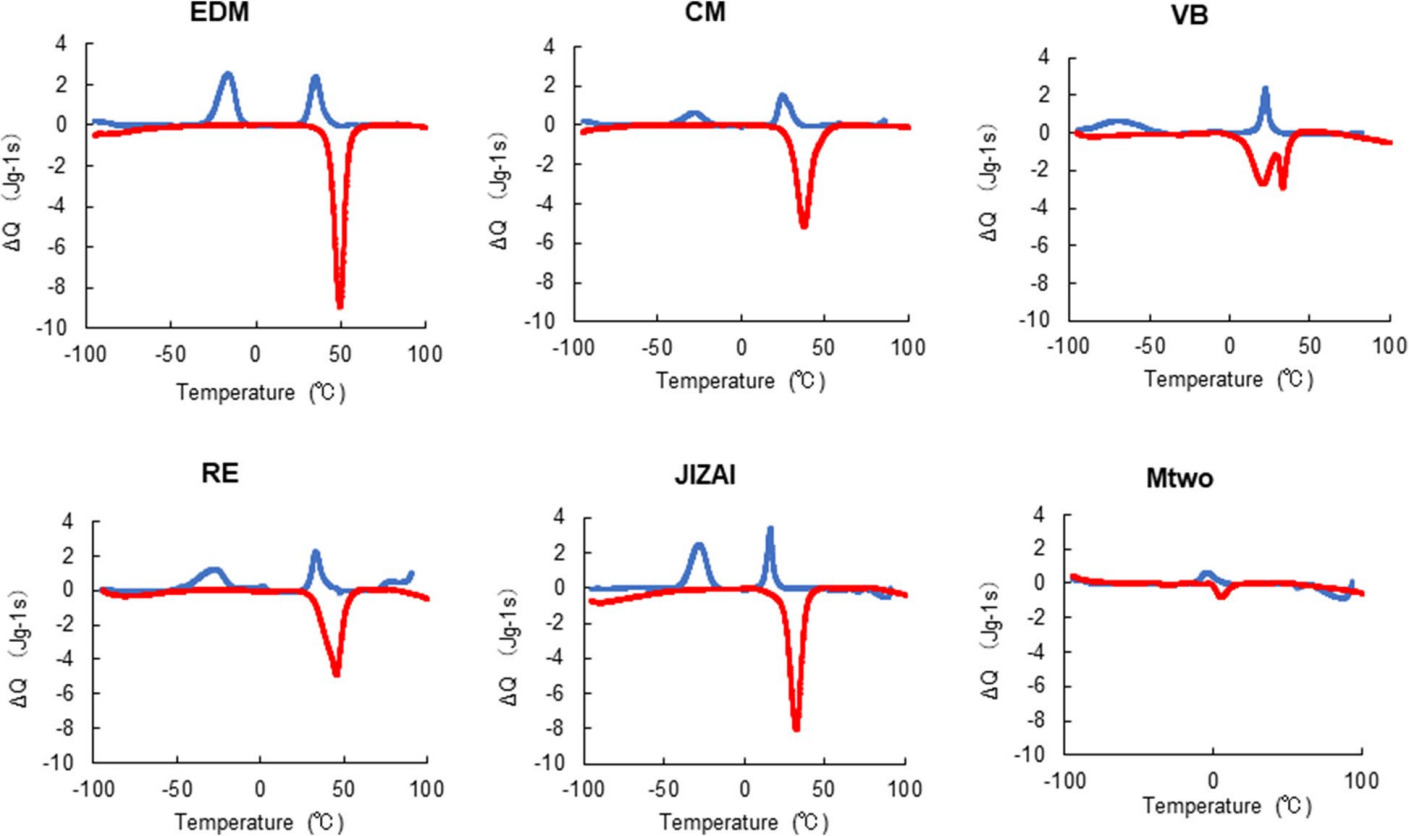
Typical differential scanning calorimetry curves for each instrument. The blue and red lines show the cooling and heating curves, respectively

Figure 2 shows typical DSC curves obtained for each instrument. The upper peak corresponds to the exothermic austenite-to-martensite transformation during the cooling process, while the lower peak indicates the endothermic reverse transformation during the heating process. The five heat-treated NiTi instruments exhibited two exothermic peaks, indicating R-phase transformation followed by martensitic transformation. VB showed two endothermic peaks during the heating process.

Table 2 shows the transformation temperatures obtained from the DSC traces. EDM showed the highest R*s*, R*f*, A*s*, and A*f* among the studied instruments. A*f* of the five heat-treated instruments were higher than or similar to BT. Mtwo showed the lowest A*f*, which was even below RT.

**Table 2:** Phase transformation temperatures

Instrument	M*s* (°C)			M*f* (°C)			R*s* (°C)			R*f* (°C)			A*s* (°C)			A*f* (°C)		
EDM	-10.7 ± 1.0			-29.9 ± 1.7			41.7 ± 0.8			30.0 ± 0.6			41.7 ± 0.5			54.7 ± 0.7		
CM	-19.7 ± 1.6			-46.0 ± 3.2			36.1 ± 1.7			20.5 ± 1.3			26.1 ± 2.1			46.4 ± 1.4		
VB	-46.8 ± 4.9			-87.3 ± 1.9			25.8 ± 0.2			17.6 ± 0.5			8.0 ± 0.6			37.0 ± 0.5		
RE	-20.5 ± 1.6			-56.3 ± 5.9			39.2 ± 0.3			29.5 ± 0.7			26.8 ± 2.2			51.8 ± 0.5		
JIZAI	-19.9 ± 0.4			-38.8 ± 0.4			19.2 ± 0.6			12.4 ± 0.7			24.0 ± 0.9			37.7 ± 1.2		
Mtwo	5.2 ± 0.7			-14.5 ± 1.8			–			–			-3.5 ± 2.5			15.7 ± 2.2		

Phase transformation temperatures (mean ± SD) of the tested systems (*n* = 5)

### Bending loads

**Fig. 3:**
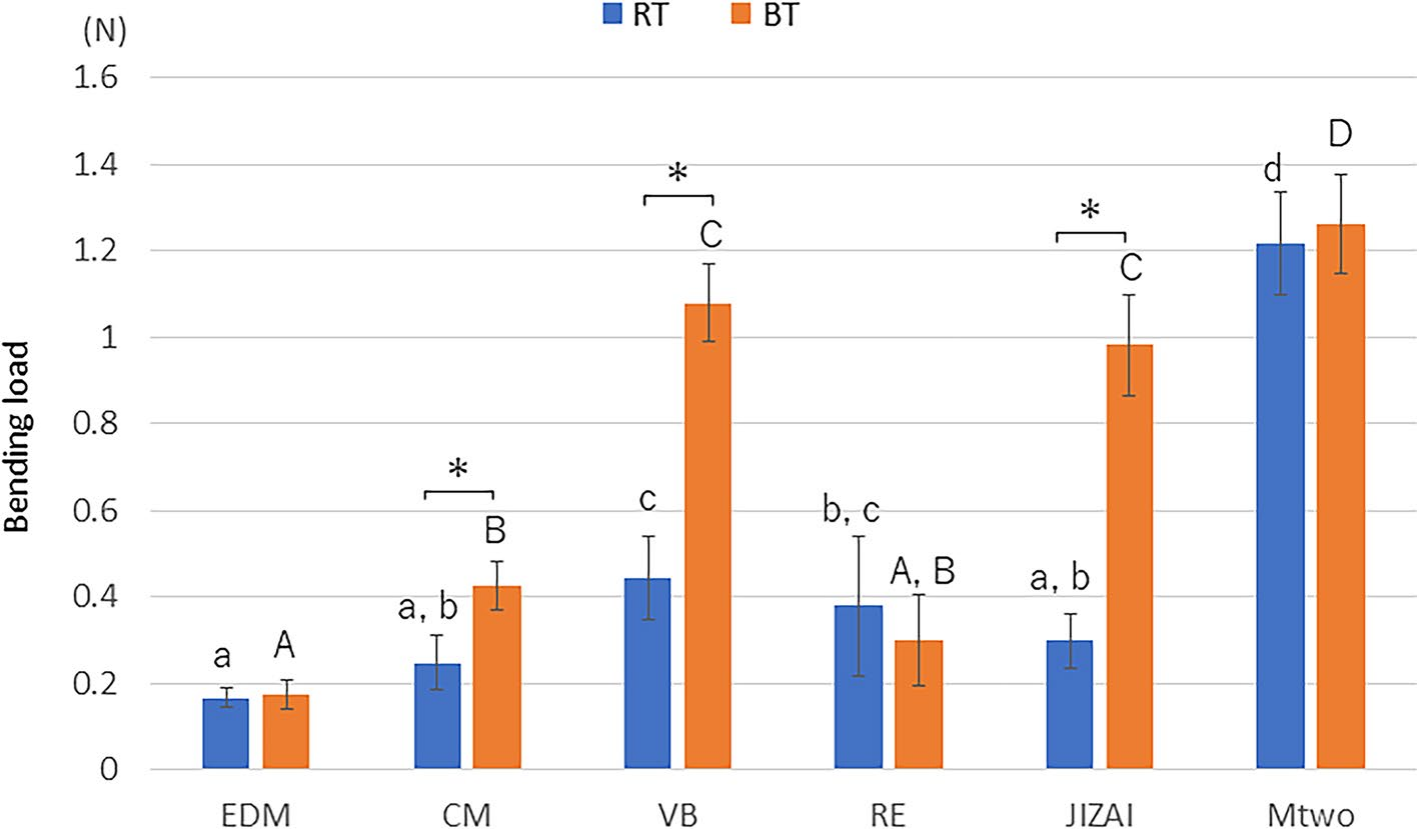
Bending load values at deflection of 0.5 mm at RT and BT (*n* = 10 in each group) Different lowercase and uppercase letters indicate that the values are significantly different between instruments at RT and BT, respectively (*p* < 0.05). * *p* < 0.05 for each instrument at RT and BT

Figure 3 shows the bending loads at a deflection of 0.5 mm at RT and BT. EDM showed the lowest bending load at both temperatures (*p* < 0.05). CM, VB, and JIZAI showed significantly higher values at BT than at RT (*p* < 0.05).

**Fig. 4:**
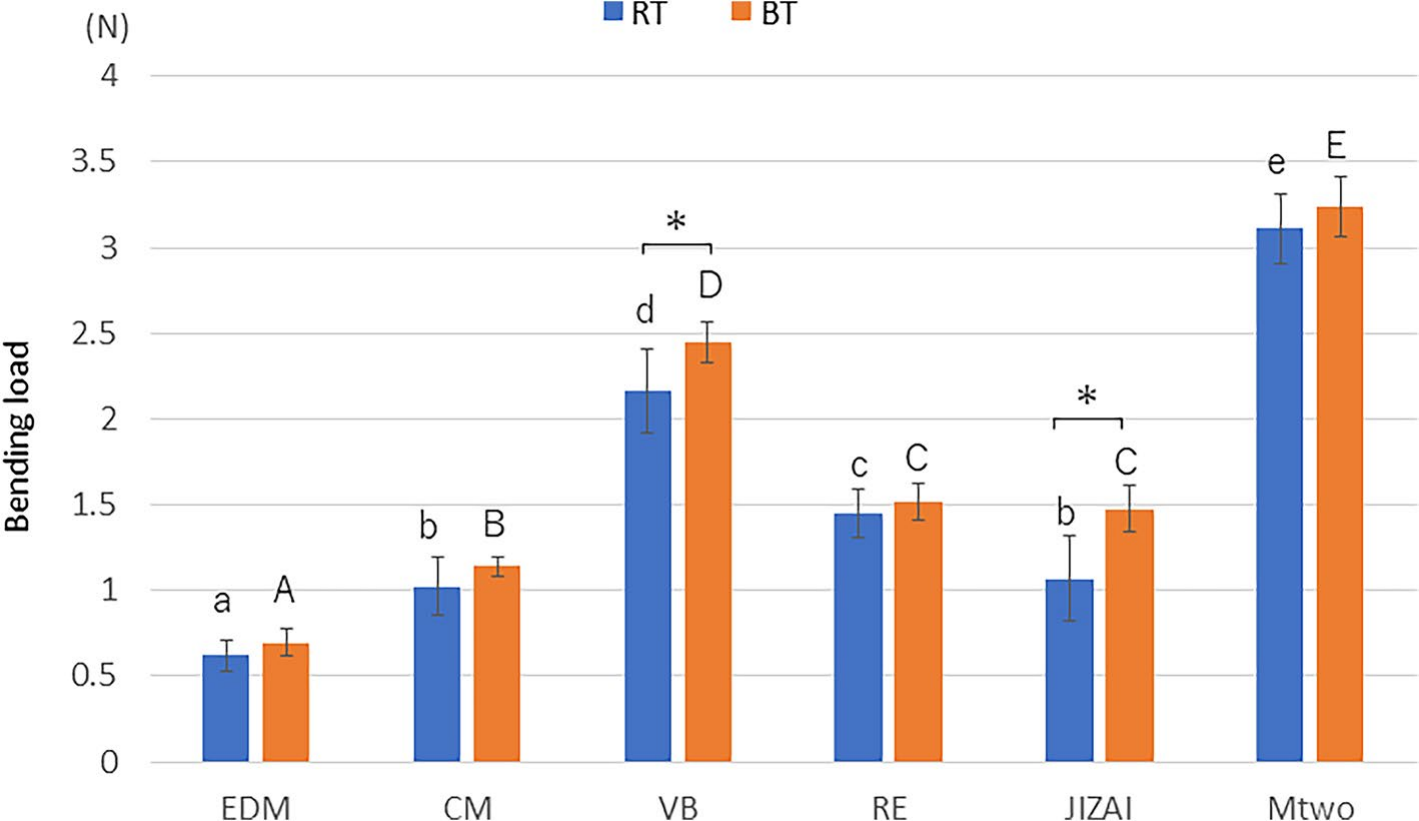
Bending load values at deflection of 2.0 mm at RT and BT (*n* = 10 in each group) Different lowercase and uppercase letters indicate that the values are significantly different between instruments at RT and BT, respectively (*p* < 0.05). * *p* < 0.05 for each instrument at RT and BT

Figure 4 shows the bending loads at a deflection of 2 mm at RT and BT. The values were in the order of EDM > CM and JIZAI > RE > VB > Mtwo at RT and EDM > CM > JIZAI and RE > VB > Mtwo at BT (*p* < 0.05). VB and JIZAI showed significant differences depending on the experimental temperature, with values higher at BT than at RT (*p* < 0.05).

### Cyclic fatigue resistance

**Fig. 5:**
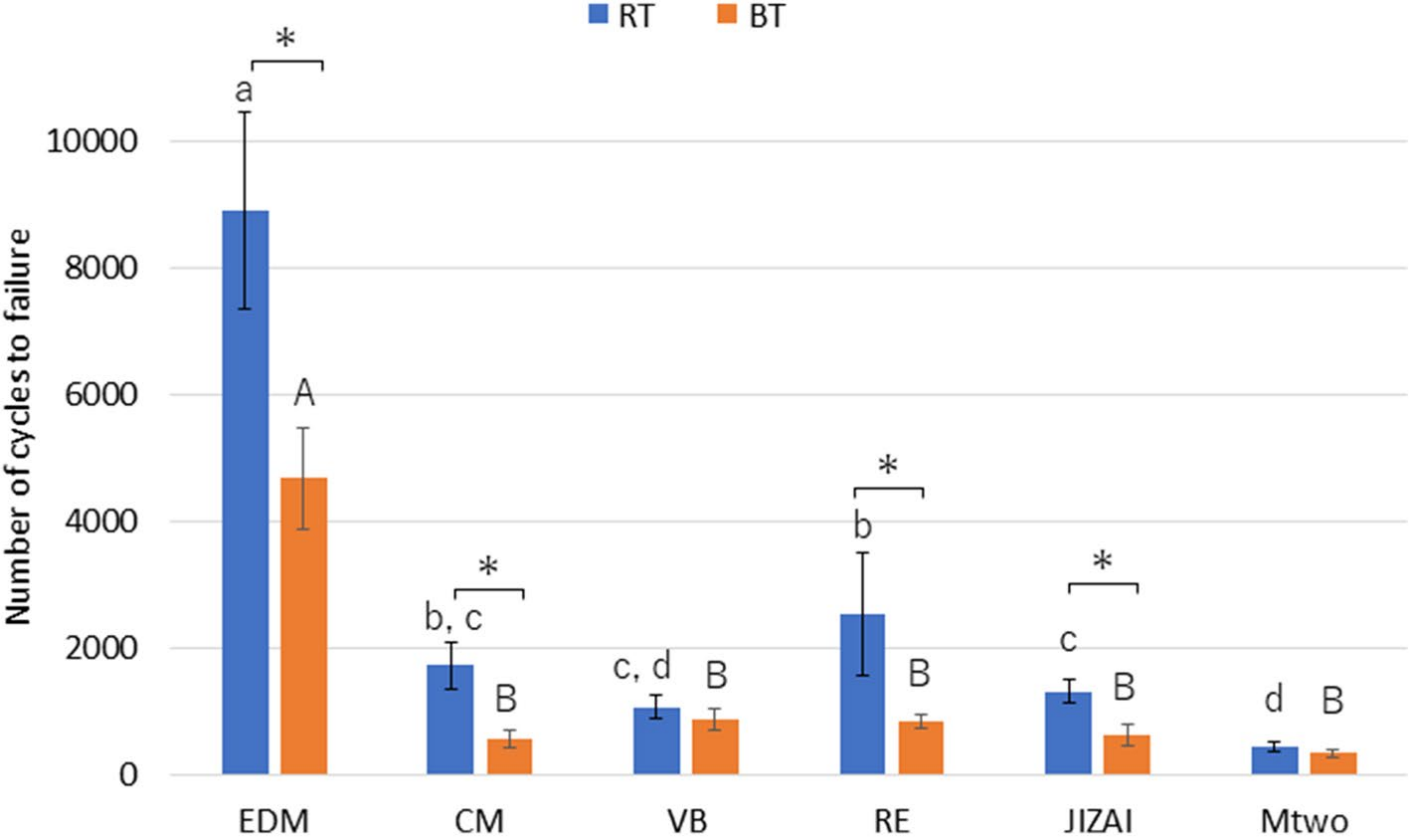
Number of cycles to failure at RT and BT (*n* = 10 in each group) Different lowercase and uppercase letters indicate that the values are significantly different between instruments at RT and BT, respectively (*p* < 0.05). * *p* < 0.05 for each instrument at RT and BT

As shown in Fig. 5, EDM exhibited the highest NCF value among the tested instruments at both temperatures (*p* < 0.05). At RT, Mtwo exhibited significantly lower NCF values than EDM, CM, RE, and JIZAI (*p* < 0.05). EDM, CM, RE, and JIZAI showed a significantly higher NCF at RT than that at BT (*p* < 0.05).

## Discussion

Phase transformation is one of the most important factors determining the mechanical properties of NiTi alloys, because NiTi alloys at different phase composition exhibit different mechanical characteristics in terms of elasticity/stiffness, ductility, hardness, fatigue resistance, and superelastic/shape memory properties [14, 15, 29]. Various factors, both intrinsic to the metal and external environmental conditions, influence the phase transformation behavior of NiTi alloys, including the ratio of Ni to Ti, temperature, stress, heat treatment, and manufacturing process [4, 6]. Thus, understanding the phase transformation status of NiTi rotary instruments, particularly under BT, is crucial for the safe and efficient clinical application of these instruments.

The results of the present study showed that A*f* and M*s* of Mtwo were lower than RT, indicating that this non-heat-treated instrument is austenitic at RT and BT. The heat-treated instruments exhibited two distinct peaks during cooling, representing a two-step phase transformation involving the formation of R-phase followed by martensite [30]. R-phase plays an important role in the instrument's behavior because it has superior flexibility and less memory strain than conventional austenite–martensite materials [31]. These properties confer R-phase instruments superior fatigue resistance [32, 33] and enhanced canal shaping ability [16]. The peak in the heating curve indicates austenite transformation (reverse transformation). VB showed two peaks during heating, indicating that R-phase is present during the reverse transformation. The A*f* of EDM was higher than BT, indicating that this instrument is primarily composed of martensite/R-phase at both RT and BT. CM, RE, and JIZAI had an A*s* that was close to RT and an A*f* that was similar to or higher than BT, indicating that these instruments are composed of a mix of martensite/R-phase and austenite at RT, and the proportion of austenite phase is greater at BT than at RT. These findings are supported by an earlier X-ray diffraction analysis at RT (25 °C), which showed that EDM has a significant amount of R-phase in addition to B19′ martensite, while CM has both austenite and martensite and a small amount of R-phase [25]. VB may also contain a mix of martensite/R-phase and austenite at both RT and BT as well as a greater proportion of austenite than the other heat-treated instruments, as suggested by the low A*s* (8.0 °C).

The bending properties of NiTi alloys differ between the elastic and superelastic ranges. The bending load in the elastic range depends on the elastic modulus of the instrument, which varies depending on the constituent phases, and the elastic modulus of the martensitic phase is lower than that of austenitic phase [14]. In the superelastic range, NiTi alloys undergo substantial reversible deformation without permanent plastic deformation or fracture [14, 15]. This property enhances the clinical application of NiTi rotary instruments through improved flexibility and enhanced ability to maintain original canal curvature [16].

The results of the bending tests showed that Mtwo exhibited significantly higher load values than the other instruments at RT and BT, indicating that heat treatment improves the flexibility of the instruments. Moreover, CM, VB, and JIZAI showed significantly higher bending loads at BT than RT. Thus, the null hypothesis on bending properties was rejected.

EDM and RE exhibited similar load values in the elastic range at BT and RT, in contrast to CM, VB, and JIZAI, which exhibited significantly higher load values at BT. This may be explained as that EDM and RE, with a higher A*f*, contain a higher proportion of martensite/R-phase at BT than CM, VB, and JIZAI.

In the superelastic range, VB and JIZAI had higher load values at BT than at RT, while EDM, RE, and CM exhibited comparable load values at both temperatures. Superelasticity arises from the reversible stress-induced martensitic transformation, and this process is influenced by the difference in the environmental temperature and A*f* [29]. The present results indicated that instruments with an A*f* higher than BT, such as EDM, RE, and CM, may require a similar level of critical stress to induce superelastic deformation at both RT and BT. These results are congruent with an earlier study showing that the bending resistance of EDM is not affected by the temperature change [21].

Cyclic fatigue tests for NiTi rotary instruments can help determine the resistance to fatigue failure of these instruments by evaluating the endurance of these instruments under repeated loading conditions [34]. There are two types of cyclic fatigue tests: static and dynamic [35, 36]. In the static test, an instrument is rotated in a root canal model without back-and-forth movement until separation, and the highest bending stress occurs at the center of the maximum root canal curvature [35, 36]. In the dynamic test, dynamic back-and-forth movement of the instrument simulates the stress that is generated on an instrument in the clinical setting because the maximum point of flexure varies along the instrument throughout the testing procedure [34]. Instruments are never used in a fixed position in clinical practice, and thus dynamic testing is considered more appropriate because it more closely reproduces actual conditions than static testing [37–39]. Therefore, dynamic cyclic fatigue test was conducted in this study.

The results of the dynamic cyclic fatigue test revealed several significant differences among the instruments and in the same instrument at the two temperatures. Thus, the null hypothesis on cyclic fatigue life was rejected. EDM, CM, RE and JIZAI showed a significantly higher NCF than Mtwo at either or both temperatures, supporting the fact that heat treatment improves the cyclic fatigue resistance of NiTi rotary instruments [16, 40] by modifying the phase transformation temperatures [4, 13, 23, 34, 35]. EDM had the highest NCF at both temperatures, which may be attributed to the finding that only EDM exhibited an A*s* higher than BT. According to a previous study comparing CM and EDM of the same size as the investigated instruments in this study (#40/0.04 taper), EDM has an approximately 700% higher NCF than CM [25]. In this study, the NCF of EDM was approximately 500% higher than that of CM, which may be attributed to the difference in the dimension of the model root canals (such as radius and degree of curvature) and the test condition, i.e., dynamic versus static. A previous study used dynamic cyclic fatigue testing to show that JIZAI (#25/0.06 taper) and EDM One File (#25/0.08 taper at the tip) have similar NCF values [16]. This is inconsistent with the current findings. Specifically, in this study, tip size- and taper-matched conditions were employed, and the results showed that EDM was more resistant to cyclic fatigue than JIZAI.

NCF values of heat-treated instruments, except VB, were lower at BT than at RT, supporting earlier studies, which showed that NiTi rotary instruments are more resistant to cyclic fatigue at the lower temperature [19, 20]. The R*f* of EDM was between BT and RT, and thus EDM is richer in R-phase at RT than at BT, which explains the decreased NCF value at BT. However, some studies have reported contradictory findings that the NCF of EDM is unaffected by the environmental temperature [19–21, 41]. The contradictory findings may be attributed to differences in instrument size (EDM One file versus #40/0.04 taper), the dimension of the model root canal, and the test condition, i.e., static versus dynamic. The decreased NCF values of CM, RE, and JIZAI at BT may be associated with the greater proportion of austenite at BT than at RT. In contrast, the NCF of VB was unaffected by the environmental temperature, although a previous study showed that the NCF of VB is lower at 37 °C than at 20 °C [20]. The present finding may be attributed to the lower A*s* of VB, indicating that VB is richer in austenite at RT, and thus the difference in phase composition at RT and BT is less prominent compared with the other heat-treated instruments.

The mechanical properties of NiTi rotary instruments are influenced by a variety of factors and are difficult to attribute to a single factor. Thus, in addition to phase composition, geometrical differences were examined since they can be crucial for determining the flexibility and cyclic fatigue resistance of NiTi rotary instruments [23]. In other words, the effect of thermal treatment on the mechanical properties of NiTi instruments can be different depending on the configuration of the instrument. In particular, smaller cross-sectional areas and core diameters are associated with increased flexibility [42] and cyclic fatigue resistance [23, 24, 43]. It is also recognized that more flexible instruments tend to exhibit greater resistance to cyclic fatigue [13]. In this study, despite the same tip size and taper, the core diameter, cross-sectional shape and pitch length differed owing to the specific design features among the instruments. Although the present DSC analysis suggested that CM is poorer in martensite/R-phase than RE, the bending tests indicated that CM is more flexible in the superelastic range than RE, which may be attributed to the smaller core diameter of CM. In contrast, VB and RE were almost the same in cross-sectional shape and area and similar in the number of blades, whereas RE exhibited a clear trend of lower bending loads and higher NCF values than VB. Thus, in determining these properties, phase compositional difference, rather than geometry, plays a major role as long as the two instruments are considered. EDM exhibited superior flexibility and cyclic fatigue resistance, which agrees with earlier findings [13, 19–21] and may be explained by its higher A*f* and smaller core diameter than the other instruments.

Collectively, the present study clearly demonstrated that, at RT, various heat-treated NiTi rotary instruments showed higher transformation temperatures and improved flexibility and cyclic fatigue resistance than Mtwo. At BT, however, instruments in a mixed status of martensite/R-phase and austenite (CM, VB, RE and JIZAI) did not show significant difference in NCF values compared to Mtwo. Furthermore, these instruments were inferior in either flexibility or cyclic fatigue resistance, or even both at BT, when compared to RT. In the clinical setting, NiTi rotary instruments usually receive a temperature change from RT to BT, starting from insertion into the canal. After 4 min of irrigation with an RT solution, the intracanal temperature is reported to reach 35 °C [44], indicating that the temperature rise may play a role in the shaping performance of heat-treated NiTi rotary instruments. At present, no evidence has been provided to indicate the extent to which instruments’ properties are affected by the intracanal temperature change. However, the present findings may support the view that testing at RT can lead to an overestimation of the heat-treatment-induced improvement in the bending properties and fatigue life of NiTi instruments [45]. The advantages derived from heat treatment may be less prominent at BT, particularly in instruments with mixed phases, which may exhibit prominent phase compositional differences at RT and BT.

Care should be taken when the present in vitro findings are extrapolated to the clinical setting owing to the following limitations of this study. First, although efforts were made to mimic clinical conditions during the dynamic cyclic fatigue tests, there could be several differences between laboratory and clinical conditions, including the canal shape, friction generation, and length and speed of pecking motion. Second, as an inherent limitation of most studies employing commercially available instruments, several confounding geometric factors other than the size and taper were not eliminated, which complicated the attribution of differences between groups to a single factor [46]. The use of pair(s) of instruments that differ in one particular parameter, such as metallurgy, may be essential to evaluate the impact of the parameter. Third, the temperature conditions (RT and BT) of the present study cannot fully simulate the actual temperature change that a NiTi instrument may receive during clinical operation. Further study is required to determine comprehensively how NiTi rotary instruments perform under more clinically relevant temperature conditions, with tests for other parameters, such as torsional resistance, cutting efficiency, torque/force generation during instrumentation, and root canal shaping ability.

## Conclusions

Within the limitations of this study, it was concluded that (i) heat-treated NiTi instruments exhibited lower bending loads and higher NCF values than non-heat-treated Mtwo and (ii) the improved mechanical properties of heat-treated NiTi instruments were less pronounced at BT than at RT, particularly in the NCF values of instruments with a mixture of martensite/R-phase and austenite phases at the tested temperatures.

## Data Availability

The datasets used and/or analyzed during the current study are available from the corresponding author on reasonable request.

## Abbreviations

RT: Room temperature
BT: Body temperature
NiTi: Nickel-titanium
EDM: Hyflex EDM
CM: Hyflex CM
VB: Vortex Blue
RE: RE file CT
DSC: Differential scanning calorimetry
M*s*: Martensitic transformation starting temperature
M*f*: Martensitic transformation finishing temperature
A*s*: Reverse transformation starting temperature
A*f*: Reverse transformation finishing temperature
R*s*: R-phase transformation starting temperature
R*f*: R-phase transformation finishing temperature
NCF: Number of cycles to failure
ANOVA: Analysis of variance
